# Monitoring of the Surface Charge Density Changes of Human Glioblastoma Cell Membranes upon Cinnamic and Ferulic Acids Treatment

**DOI:** 10.3390/ijms21186972

**Published:** 2020-09-22

**Authors:** Monika Naumowicz, Magdalena Kusaczuk, Marcin Zając, Miroslav Gál, Joanna Kotyńska

**Affiliations:** 1 Laboratory of Bioelectrochemistry, Department of Physical Chemistry, Faculty of Chemistry, University of Bialystok, K. Ciolkowskiego 1K, 15-245 Bialystok, Poland; mzajc@wp.pl (M.Z.); joannak@uwb.edu.pl (J.K.); 2 Department of Pharmaceutical Biochemistry, Medical University of Bialystok, Mickiewicza 2A, 15-222 Bialystok, Poland; mkusaczuk@wp.pl; 3 Department of Inorganic Technology, Faculty of Chemical and Food Technology, Slovak University of Technology, Radlinského 9, 812 37 Bratislava, Slovakia; miroslav.gal@stuba.sk

**Keywords:** cinnamic acid, ferulic acid, phenolic compounds, LN-229 cells, glioblastoma, chemoprevention, microelectrophoretic mobility measurements, surface charge density, quantitative characteristics, pH

## Abstract

Cinnamic acid (CA) and ferulic acid (FA) are naturally occurring phenolic acids claimed to exert beneficial effects against disorders related to oxidative stress, including cancer. One such malignancy that still remains a therapeutic challenge mainly due to its heterogeneity and inaccessibility to therapeutic agents is *Glioblastoma multiforme* (GBM). Here, the influence of CA and FA on the surface charge density of human GBM cell line LN-229 was studied using the electrophoretic light scattering technique. Also, the cytotoxicity of both phenolic acids was determined by metabolic activity-assessing tetrazolium test (MTT) analysis after exposure to CA and FA for 24 h and 48 h. Results showed that both compounds reduced cell viability of LN-229 cells, with more pronounced effect evoked by CA as reflected in IC_50_ values. Further analyses demonstrated that, after treatment with both phenolic acids, the negative charge of membranes decreased at high pH values and the positive charge of the membranes increased at low pH values compared to the data obtained for untreated cells. Afterward, a four-equilibrium model was applied to estimate the total surface concentrations of both acidic and basic functional groups and their association constants with solution ions in order to calculate theoretical values of membrane surface charge densities. Then, the theoretical data were compared to the experimental data in order to verify the mathematical model. As such, our results indicate that application of electrochemical methods to determine specific drug–membrane interactions might be crucial for predicting their pharmacological activity and bioavailability.

## 1. Introduction

Naturally occurring phenolic acids and their analogues present potentially beneficial effects to human health in terms of numerous oxidative stress-related diseases including cancer. Abundant in vitro and in vivo studies have shown that, thanks to their antioxidant properties, these compounds exhibit anticancer potential mainly through disruption of cell grown and metastasis and stimulation of cancer cell death. Preclinical investigations have revealed that using a combination of phenolic acids with traditional chemoradiotherapy or other polyphenols may be potentially efficient in reducing the spread of cancer [1]. In recent years, the cinnamic acid and its derivatives such as coumaric, caffeic, and ferulic acids have attracted considerable attention not only due to their pharmaceutical [2] and biological [3] activities but also to their technological and industrial applications [4].

Cinnamic acid and its derivatives may be considered as weak organic acids [5]. It has been demonstrated that their biological characteristics such as transport, distribution, selectivity, activity, and toxicity is at least partially determined by their interaction with biological membranes [6]. These compounds are sometimes able to interact with/pass through the biological membranes [7], but such ability depends largely on substitutes contained in their main structure. Therefore, compounds with a comparable structure frequently interact with biological membranes in a different way, owing to the complex correlation (which can be modified by pH changes) between permeability and liposolubility, which can be further modified by pH changes [8].

Lipophilicity is known as a first-rate physicochemical parameter of chemical compounds that affects their biological activity. Lipophilicity is defined by the partitioning of a compound between an aqueous and an organic (octanol) phase, and a partition coefficient (log*P*) is used for this characterization. The log*P* is determined for an uncharged drug species, and its value is usually in the range from −3 (very hydrophilic) to +10 (extremely hydrophobic) [9]. The more lipophilic the compound, the greater its ability to pass through the lipidic bilayer of the cell membrane and thus the higher its bioavailability. However, in the case of excessively lipophilic compounds, the problem is their limited solubility in the water of the organism, so they are not properly absorbed [10]. This makes determination of liposolubility an important estimator of the bioavailability of potential drugs in modern pharmacology.

Since the most common and yet least invasive method of drug application is oral administration, a large number of predictive models have been developed to establish the potential oral bioavailability of tested compounds. Most of these models are based on a number of physicochemical descriptors. One of the most important and most applied rules concerning this issue is Lipinski’s Rule-of-Five (Ro5) [11]. Ro5 advocates that molecules with weak oral permeability and absorption have two or more of the following attributes: molecular weight (*M*_WT_) greater than 500, calculated n-octanol/water partition coefficient (*c*log*P*) greater than 5, more than five hydrogen bond donor groups (HBD) (expressed as the sum of OHs and NHs groups), and more than 10 acceptor groups (HBA) (expressed as the sum of Os and Ns atoms).

Experimental and theoretical studies of the electrical properties of bilayer lipid membranes, both natural and artificial, have greatly contributed to our knowledge of the properties, function, and structure of cell membranes. The surface charge of membranes is an extremely important electrical parameter that depends on the composition of the cytoplasmic membrane and the physiological condition of cells. This parameter determines the electrostatic interactions between a solute and a membrane, and these interactions have been reported to play an important role in binding drugs to the membrane [12]. The cell-surface charge can be quantified by measuring electrokinetic potential (zeta potential), which characterizes the electrical double-layer potential on the cell surface. Zeta potential varies according to many parameters, such as temperature, pH, conductivity (ionic strength), and solvent (viscosity). Thus, small modifications to any of these parameters may have a significant influence on the zeta potential values [13]. One of the techniques that might be used to estimate zeta potential is the electrophoretic light scattering (ELS) technique based on dynamic light scattering (DLS), in which the shift in the frequency or in the oscillation phase of the laser beam depends on the mobility of particles/cells in an alternating electric field [14]. In this respect, in our previous works, we have already applied the ELS technique to study human glioblastoma cells treated with either silica nanoparticles [14,15] or phenolic compounds such as quercitin [16] and p-coumaric acid [17,18].

The major aim of the present paper is to investigate the effect of cinnamic acid (CA) and its hydroxy derivative—ferulic acid (FA)—on the surface charge density of human glioblastoma multiforme (GBM) cell membranes using the ELS technique. The selected compounds are common phenolic antioxidants occurring naturally in or being added to food. These phenol acids present structural similarity to each other, so the determination of the correlation between their structures and their surrounding solutions seems reasonable approach in evaluating differences in their molecular effects. Chemical formulas of selected polyphenols are presented in Table 1. Existing reports already show considerable amount of data on the hydroxycinnamic acids activity in various types of malignancies, but only limited information is available concerning CA efficiency in brain tumors (Table 1). More importantly, no such evidence exists in terms of FA in GBM cells. This makes analysis of physicochemical properties of CA and FA in GBM cells an important issue to study in order to determine potential drug-membrane interactions and predict oral availability of these compounds.

Although much is already known about the pathology and therapy of GBM, it is still impossible to efficiently treat this malignancy [30,31]. One of the reasons of this failure is that, in contrast to other cancers, brain tumors are extremely inaccessible to chemotherapeutics because of the blood-brain barrier (BBB) and a number of additional factors impairing the effectiveness of available treatments [32]. Thus, glioblastoma still represents a therapeutic challenge with poor overall prognosis.

Current experimental therapeutic strategies strongly rely on the utilization of natural compounds in cancer chemoprevention/chemotherapy. Therefore, the presented paper is focused on evaluating the influence of CA and FA (its derivative) on the physicochemical and electrical parameters of the GBM cell membranes. As such, although preliminary these results might give a valuable information on the drug-membrane interactions and establish novel standard of fundamental analyses concerning drugs bioavailability in modern pharmacology.

## 2. Results

Deep understanding of the influence of phenolic acids on the electrical and physicochemical properties of cell membranes is utterly essential, in terms of many potential applications in membrane science. In the past few years*,* multiple studies have shown the strong potential of cinnamic acid and its derivatives in inhibiting the growth of various cancer cells in vitro. However*,* there is limited data available on the influence of these compounds on the electrical properties of cell membranes. Also, the lack of quantitative descriptions of equilibria existing in membranes is a serious obstacle to overcome while trying to estimate membrane interaction with a particular compound.

We therefore investigated whether cinnamic and ferulic acids could contribute to the changes in the values of electrical parameters of cancer cell membranes.

### 2.1. The Influence of CA and FA on Cell Viability

**Figure 1:**
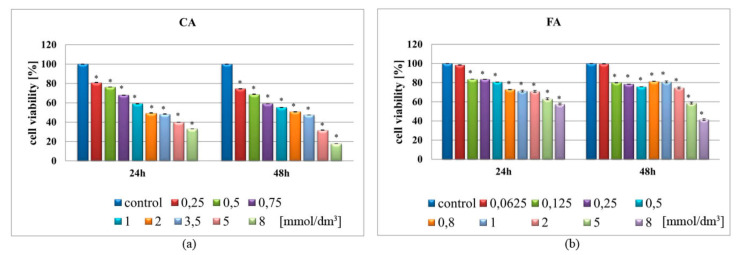
Effect of cinnamic acid (CA) and ferulic acid (FA) on the viability of human glioblastoma LN-229 cells. Cells were treated for 24 h and 48 h with the indicated concentrations of CA (**a**) and FA (**b**). The viability of cells was determined using the metabolic activity-assessing tetrazolium test (MTT) assay. The results represent means for pooled triplicate values from three independent experiments. * indicates statistical significance (*p*-value < 0.05) when comparing to untreated control cells.

First, in order to check if both cinnamic and ferulic acids were able to reduce viability of LN-229 cells, a metabolic activity-assessing tetrazolium (MTT) test was performed. Despite certain limitations of this analytical assay, the MTT test is routinely utilized as screening tool for cytotoxicity of potential drugs [33]. As shown in Figure 1, treatment of LN-229 cell line with either CA and FA resulted in a dose- and time-dependent decrease of cell survival. Both acids significantly diminished the proliferation rate of GMB cells; however, CA presented more pronounced effects, reaching almost 80% of inviable cells in the highest tested concentration after 48 h of treatment (Figure 1a). Less pronounced effects were observed for FA, where the highest used concentration (8.0 mmol/dm^3^) reduced viability of LN-229 cells up to 40% after 48 h (Figure 1b). Higher cytotoxicity of CA was also confirmed by the calculated IC_50_ values being 1.623 mmol/dm^3^ and 5.019 mmol/dm^3^ for CA and FA, respectively (calculated after 48 h of treatment). Dose-response curves are demonstrated in the Supplementary Figure S1. Based on the MTT results, two concentrations of CA (1.0 and 3.5 mmol/dm^3^) and FA (1.0 and 5.0 mmol/dm^3^) were selected to proceed for further electrochemical analysis.

### 2.2. The Influence of CA and FA on Surface Charge Densities of Cell Membranes

**Figure 2:**
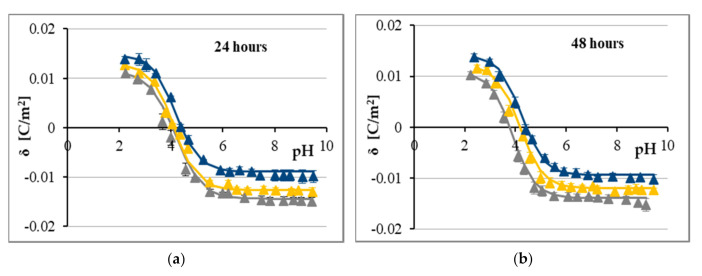
Surface charge density of LN-229 cell membrane versus pH of electrolyte solution. The cells were untreated (grey) or treated with 1.0 (yellow) and 3.5 (navy blue) mmol/dm^3^ of CA for 24 h (**a**) and 48 h (**b**). Points denote the experimental values, and the continuous line links the theoretical values.

**Figure 3:**
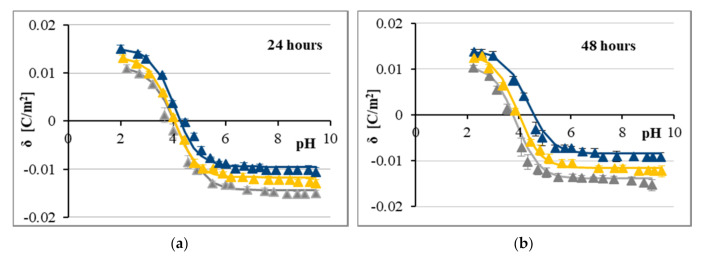
Surface charge density of LN-229 cell membrane versus pH of electrolyte solution. The cells were untreated (grey) or treated with 1.0 (yellow) and 5.0 (navy blue) mmol/dm^3^ of FA for 24 h (**a**) and 48 h (**b**). Points denote the experimental values, and the continuous line links the theoretical values.

According to the MTT results and following the calculation of the IC_50_ values, CA showed higher cytotoxicity to LN-229 cells than FA. Although various causes may underlie these discrepancies in the cytostatic effect, one of the plausible reasons of such differences might be the distinct pattern of drug-membrane interaction. Thus, the microelectrophoretic mobility measurements were performed for LN-229 cells treated with CA (Figure 2) and FA (Figure 3). Analyses were performed at pH values between 2 and 10, using sodium chloride as the supporting electrolyte. The experimental values of the surface charge densities were calculated from electrophoretic mobility according to Equation (1) and are shown as points. The theoretical values of the surface charge densities were obtained on the basis of Equation (3) and are presented as curves on all figures.

According to Figure 2 and Figure 3, the dependencies of surface charge density on pH in analyzed LN-229 cell membranes produced similarly shaped curves regardless of the treatment conditions. Along with the decrease in pH values, the increase in positive surface charge density was observed, but only up to a certain point. Conversely, with the increase in pH values, the negative charge of membranes increased until the plateau was reached. The current results demonstrated that treatment of LN-229 cells with either CA or FA resulted in noticeable alterations of surface charge densities of the aforementioned membranes—the negative charge of membranes decreased at high pH values and the positive charge of the membranes increased at low pH values compared to the data obtained for untreated cells (Figure 2 and Figure 3). These alterations seemed to be dose-dependent but not time-dependent. Thus, although both studied acids visibly modulated the surface charge values of LN-229 cell membranes, no evident-enough difference between CA and FA was observed to definitively state stronger charge-modulatory effect of any of those agents.

Finally, based on the results presented in Figure 2 and Figure 3, it can be concluded that the isoelectric point of GBM cell membranes treated with both phenolic acids shifted to higher pH values compared with untreated cell membranes.

In addition to the experimental points, solid lines referring to the four equilibria model (Theory section) are plotted in Figure 2 and Figure 3. Adaptation of the model characterizing the adsorption of electrolyte ions on the cell membrane surface enabled the quantitative determination of the membrane parameters. The total concentrations of functional acidic (*C*_TA_) and basic (*C*_TB_) groups on membranes of treated and untreated LN-229 cells, as well as their average association constants with hydrogen (*K*_AH_) and hydroxyl (*K*_BOH_) ions, were calculated according to Equations (4) and (5). Calculated *C*_TA_, *C*_TB,_ *K*_AH_, and *K*_BOH_ values were introduced into Equation (3) in order to obtain theoretical data of surface charge densities for analyzed GBM cell membranes at different pH values. Figure 2 and Figure 3 show the results of this procedure (solid lines) in relation to the experimental data (triangle points). The close agreement between the calculated (theoretical) and experimental results justifies the validity of the approach.

The values of physicochemical parameters characterizing LN-229 cell surface (*C*_TA_, *C*_TB,_ *K*_AH_, and *K*_BOH_) are collected in Table 2. Data were analyzed as means with standard deviation using standard computational analysis. In line with these data, it is evident that treatment of LN-229 cells with CA and FA changes the values of physicochemical parameters of cell membranes. Of note, both the values of surface concentration of *C*_TB_ groups and the association constants of negatively charged *K*_AH_ groups increased, whereas the surface concentration of *C*_TA_ groups as well as the association constants of positively charged *K*_BOH_ groups decreased.

## 3. Discussion

Gliomas are malignant tumors of glia, the brain connective tissue. They are ranged from the least severe grade I to the most severe grade IV. *Glioblastoma multiforme* is the most aggressive and deadliest type of primary brain tumors in adults. Despite advances in surgery, radiotherapy, and chemotherapy, patients often survive only 12–18 months following diagnosis [34,35]. The human brain consumes 20% of the total body oxygen [36] and therefore is greatly susceptible to alterations in oxygen supply. Exposure of the brain to oxidative stress is one of the most important risk factors for GBM [37]. It can be alleviated by intake of antioxidants originated from plants, and therefore, these compounds are tested for their protection of brain functions [38]. Unfortunately, the results of the studies conducted so far seem to be disputable, as most of the examined compounds do not have any therapeutic prevalence. Indeed, there is a significant discrepancy between pre-clinical and clinical trial results. This may be connected not only with the design of clinical trials but also with the pharmacokinetics of the antioxidants being evaluated. In fact, some of them do not have the ability to spread effectively through biological barriers, in particular the blood–brain barrier (BBB), hindering their activity at target sites in the central nervous system.

According to Rossi et al. [39], the most important property of food polyphenols to protect the brain is their ability to cross the BBB. For example, curcumin is highly lipophilic and might pass the BBB and reach the brain, but its bioavailability is very low [40]. Therefore, in order to better understand the aspect of application of phenolic compounds in brain health, it is very important to know the fate of these compounds in the human body. The bioavailability of phenolic compounds varies greatly depending on their chemical structure, metabolism and biological activity [41].

Selection of the compounds for the study was done based on their pharmacokinetic requirements to cross the blood–brain barrier and to display relatively good bioavailability. The trans form of cinnamic acid was chosen for its ubiquitous distribution in nature in comparison to less abundant cis isomer, whereas ferulic acid was selected as a CA derivative containing additional methoxy and hydroxyl substitutes in three and four positions on the phenyl ring, respectively.

Regarding the importance of physiochemical properties of potential candidates for drugs, we checked whether CA and FA fulfill the desired criteria of the Lipinski’s ”rule of five” which is a rule of thumb to determine if a chemical compound with certain pharmacological or biological activity has properties that would make it a likely orally active drug in humans. Both tested compounds obeyed the Ro5 with *M*_WT_ < 500, *c*log*P* values < 5, number of HBA < 10, and number of HBD < 5, as shown in Table 3. Therefore, these molecules show desirable drug-like properties which encourage their further testing on the molecular and cellular levels, to use as potential pharmacotherapeutic agents in GBM treatment.

Several factors regulate the ability of a molecule to penetrate the BBB—namely, lipophilicity, molecular weight, number of hydrogen bond donors and acceptors, polar surface area, and molecular flexibility [11,42]. Lipophilicity is directly attained by *c*log*P*, and it was theoretically indicated that optimal BBB penetration occurs when the *c*log*P* values are in the range of 1.5–2.7 [42]. The *c*log*P* value for CA is 1.98, which is within this range; however, for FA, the *c*log*P* is equal 1.42 and is slightly below the range values (Table 3). The polar surface area and molecular flexibility could be determined by the number of rotable bonds (RBs), and this physicochemical parameter should be less than/or equal to 10 [43], which is fulfilled by both tested compounds. The optimal values for other factors that regulate the ability of a molecule to penetrate the BBB, which are the molecular weight of the compound and a number of hydrogen bond donors and acceptors, have been established in the modified version of the Ro5. According to this version of Lipinski’s rule to penetrate the central nervous system, the physical parameters of the compound display smaller ranges of values of than those required for general therapeutics: *M*_WT_ ≤ 400, *c*log*P* values ≤ 5, number of HBA ≤ 7, and number of HBD ≤ 3 [42]. The values of selected physicochemical parameters describing drug-like activities of CA and FA are collected in Table 3.

Based on the data collected in Table 3, we found that cinnamic acid as well as ferulic acid seem to be compounds with the ability to cross the BBB and, thus, may gain attention as plausible therapeutic targets in GBM treatment. Also, both CA and FA have been demonstrated to inhibit proliferation of various cancer cells, as exemplified in Table 1. Although for FA there are yet no results demonstrating cytostatic effect in GBM cells, such activity has already been confirmed in other types of malignancies, such as breast, lung or colon cancer (Table 1). Since the uncontrolled cellular proliferation is a hallmark of cancer cells, the efficiency of anticancer drugs can be measured by their ability to attenuate the viability of cells [46]. Thus, the cytotoxicity of both phenolic acids was evaluated by the MTT method (Figure 1). As expected, the cellular viability of CA and FA-stimulated LN-229 cells decreased when compared to the control groups. These results indicate that these compounds possess potential cytostatic activity against GBM cells.

Next, we utilized the ELS technique to extract a set of electrical-based parameters corresponding to the cancer cell state. The electrical properties of each membrane of living cells are determined by acid-base and complex formation equilibria. Both the components of membranes including proteins, phospholipids and fatty acids and the environment surrounding the membrane contribute to these equilibria [47]. Regarding the fact that the major part of electric properties in cells is dependent on cell membranes and cell composition [48], it is reasonable to expected possible changes in these properties in a cancer cells. One such change is a reduced transmembrane potential of cancer cells due to increased electronegativity of the extracellular surface caused by the alterations at the cell membrane [49]. Furthermore, the permeability of cancer cells is impaired, which changes their intracellular ionic composition when compared to normal cells. It has been reported that cancer cells are characterized by higher concentrations of sodium and chlorine [50] and lower concentrations of potassium, zinc, calcium and magnesium. Additionally, water content in transformed cells was reported to be significantly enhanced [51]. Alterations in cell membrane composition in cancer cells are associated with increased content of: sialic acid [52], free fatty acids, acidic/basic functional groups and phospholipids, and a decrease in the integral membrane proteins level [53]. It has also been known that cancer cells have a disturbed pH profile because their extracellular space is usually acidic, whereas the intracellular environment is alkaline, as opposed to normal cells [54]. Moreover, it has been stated that the cell membrane charge increases during tumorigenesis and decreases during necrosis [55]. These premises encouraged us to investigate factors influencing the electrical charge of membranes of LN-229 glioblastoma cells treated with phenolic acids. These factors include determination of the pH, evaluation of concentration of acidic and basic functional groups and also, their average association constants with hydrogen or hydroxyl ions. Thus, we determined both experimental and theoretical values of surface charge density of the cell membrane measured as a function of pH (Figure 2 and Figure 3). It is noteworthy that theoretical and experimental results are in line with each other within the analyzed pH range (between pH 2 and pH 10). As such, below and above this range, cells might undergo cell death, which is typical for normal and cancer cells [16,56]. LN-229 cells treated with either CA or FA showed an increase in positive charge at low pH and a decrease in negative charge at high pH in comparison to untreated cells. These results seem to be in agreement with other reports demonstrating similar tendency for various cancer cell membranes treated with chemotherapeutic agents [18,57,58]. It has already been well documented that polyphenols exhibit affinity for the lipid bilayer by binding to the lipid head groups near the bilayer surface (adsorption) and penetration into the bilayer interface (absorption). These compounds are able to form hydrogen bonds with membranes. They interact with phenolic hydroxyl groups acting as the hydrogen bond donors and oxygen atoms on the phospholipid acting as the hydrogen bond acceptors [59]. In line with these results, CA and FA may supposedly remain anchored to the membrane surface at least partially across the whole analyzed pH range.

The values of the parameters describing the equilibria between membrane surface components and the surrounding ions determined for untreated cells are different in comparison to the cells incubated with CA or FA (Table 2). This might be caused by the appearance of new functional groups and/or the disappearance of existing ones during the reaction of tested compounds with GBM membrane surface components. The same tendency was observed for each of the two concentrations of CA (1.0 mmol/dm^3^ and 3.5 mmol/dm^3^) and FA (1.0 mmol/dm^3^ and 5.0 mmol/dm^3^) and for cells incubated for both 24 h and 48 h. The obtained *C*_TB_ and *K*_AH_ values for a GBM cell membranes were found to be affected by tested phenolic acids and were higher than the same parameters assayed in untreated cells. Simultaneously, *C*_TA_ and *K*_BOH_ was found to be decreased in cancer cells upon CA and FA treatment. Our results seem to be in alignment with other reports, where the same trend in modulation of the values of these physicochemical parameters characterizing cancer cell surface after chemotherapeutic agents treatment was demonstrated [57,58].

Despite the plethora of data concerning anticancer activity of phenolic acids, studies report diverse efficacy of these compounds dependent on cancer type or even cell line used. The differences are related to the variations in the structure of these compounds as well as to their molecular targets. Examinations of the relationship between activity and structure demonstrate that phenolic compounds display their anticancer potential thanks to the contribution of aromatic rings and hydroxyl groups [60]. The structure of the ferulic acid differs from that of the cinnamic acid by the presence of one -OCH_3_ and one -OH group attached at the third and fourth carbon atoms of the benzene ring. It has been shown that compounds with a larger number of hydroxylic groups displayed better anticancer activity than those without hydroxyl moieties or compounds with methoxy substituents [60]. This seems to be in line with our results showing that CA is more potent than FA in preventing cancer cell proliferation (Figure 1). On the other hand, co-existence of both the preferred hydroxyl group and the undesirable methoxy group in the FA structure might be the reason why we did not notice any difference between the two studied phenolic acids in their affinity to the cell membrane surface and their influence on the values of the physicochemical and electrical properties of LN-229 cell membranes. Of note, one should keep in mind that each cell membrane has a different selectivity, that depends on its composition, which will modulate the interaction of the substances with both the inside and the surface of the membrane. Therefore, in order to clearly define the influence of both compounds on glioblastoma cell membranes, studies with broader spectrum of GBM cell lines ought to be performed.

Although phenolic compounds are extensively tested for inhibition of cancer cell growth in vitro and in vivo, there are still many aspects of their behavior that require in-depth analysis. Although naturally existing phenolic compounds are less toxic than many synthetic and semi-synthetic substances [61], at higher concentrations they can damage healthy cells. Accordingly, cinnamic acid and its derivatives are effective only at relatively high doses (about 10–20 mg/kg body weight), so dose and toxicity reduction strategies seem to be particularly important aspects of future investigations concerning these compounds [46].

Altogether, further experimental data are needed to address the many as yet unraveled issues connected with polyphenols’ function in cancer cells. Given this, differences in response to anticancer treatment in people who follow a diet rich in phenolic acids and the reasons of their insufficient therapeutic effectiveness are still to be established.

## 4. Materials and Methods

### 4.1. Reagents

DMEM containing glucose at 4.5 mg/cm^3^ (25 mmol/dm^3^) with GlutaMax, streptomycin, penicillin, and trypsin-EDTA were provided by Thermo Fisher Scientific (Waltham, MA, USA). The methylthiazolyldiphenyl-tetrazolium bromide (MTT), trans-cinnamic acid (≥98.0%; CAS No. 140-10-3), and trans-ferulic acid (99%; CAS No. 537-98-4) were provided by Sigma-Aldrich (St Louis, MO, USA).

#### Cell Culture and MTT Assay

LN-229 cell line was cultured as described in our previous works [14,32]. Briefly, cells were grown in DMEM containing 10% fetal bovine serum, 4.5 mg/cm^3^ glucose, 100 μg/cm^3^ streptomycin, 100 U/cm^3^ penicillin, and 2 mmol/dm^3^ L-glutamine and kept in an incubator (humidified atmosphere, 5% CO_2,_ 37 °C). Cells reaching confluence were seeded into 96-well plates (Nunclone) in a density of 1000 cells/well and growth medium was substituted with DMEM containing various concentrations of CA (0.25–8.00 mmol/dm^3^) or FA (0.0625–8.00 mmol/dm^3^) and further incubated for 24 h and 48 h. After incubation, cell viability was estimated using the metabolic activity-assessing tetrazolium test (MTT assay) performed according to the method of Carmichael et al. [62] described in detail in our previous work [16].

### 4.2. Methods

#### 4.2.1. Microelectrophoretic Mobility Measurements

Measurement of electrophoretic mobility of the cells was performed by Zetasizer Nano ZS system (Malvern Instruments, Malvern, UK) using the ELS technique. Experiment was carried out as a function of pH. The samples were suspended in 0.9% NaCl solution and titrated to the desired pH with HCl or NaOH. The reported values represent the average of six measurements at a given pH value.

#### 4.2.2. Experimental Surface Charge Density Determination

Based on electrophoretic mobility values, the surface charge density *δ* was determined according to following equation [63]:

(1) $$\delta =\;\frac{\eta \cdot u}{d}$$

in which: *η*—the viscosity of the solution, *u*—the electrophoretic mobility, *d*—the diffuse layer thickness.

The diffuse layer thickness was determined using the following formula [64]:

(2) $$d=\;\sqrt{\frac{\varepsilon \cdot \varepsilon_{o}\cdot R\cdot T}{2\cdot F^{2}\cdot I}}$$

in which *R* is the gas constant, *T* is the temperature, *F* is the Faraday constant, *I* is the ionic strength of 0.9% NaCl, and *ε* and *ε*_0_ refer to the permeability of the electric medium.

#### 4.2.3. Theoretical Surface Charge Density Determination

The dependence of the surface charge density of the GBM cell membrane on the pH of the electrolyte solution may be characterized using the four-equation model. Two of these equations are used to describe the equilibrium of the association of negative groups with the hydrogen and sodium ions. The other two equations concern the equilibrium of the association of positive groups, e.g., phospholipids or proteins, with the hydroxide and chloride ions. The model has been described in detail before [65], and only the final equations are given here.

After solution of the equations system, the following dependences are derived:

- the equation describing surface charge density of the cell membrane is
  (3) $$\frac{\delta }{F}=\;\frac{C_{TB}\cdot a_{H^{+}}}{a_{H^{+}}\cdot \left(1+K_{BCl\;}\cdot a_{Cl^{-}}\right)+\;K_{BOH}\cdot K_{W}}-\frac{C_{TA}}{K_{AH}\cdot a_{H^{+}}+\;K_{ANa}\cdot a_{Na^{+\;}}+1}$$
- the equations obtained by simplifications of Equation (3) to a linear form at low H+ and high H+ ion concentrations are
  (4) $$\frac{\delta }{F}\cdot a_{H^{+}}^{-1}=\;\frac{-C_{TA}\cdot a_{H^{+}}^{-1}}{1+K_{ANa\;}\cdot a_{Na^{+}}}+\left(\frac{C_{TB}}{K_{BOH}\cdot K_{W}}+\frac{K_{AH}\cdot C_{TA}}{{\left(\;1+K_{ANa}\cdot a_{Na^{+}}\right)}^{2}}\right)$$
  (5) $$\frac{\delta }{F}\cdot a_{H^{+}}=\frac{C_{TB}}{1+K_{BCl}\cdot a_{Cl}}\cdot a_{H+}\cdot \left(\frac{K_{BOH}\cdot K_{W}\cdot C_{TA}}{{\left(1+K_{BCl}\cdot a_{Cl}\right)}^{2}}+\frac{C_{TA}}{K_{AH}}\right)$$
  in which
  *σ* is the surface charge density;
  *a*_H+_, *a*_Na+_, *a*_OH−_, *a*_Cl—_are the volume concentrations of solution ions;
  *C*_TA_ is the total surface concentration of the membrane acidic groups;
  *C*_TB_ is the total surface concentration of the membrane basic groups;
  *K*_AH_, *K*_ANa_, *K*_BOH_, *K*_BC_ are associations constants;
  *F* is the Faraday constant.

The coefficients obtained from the linear regression can be applied to calculate *K*_AH_, *K*_BOH_, *C*_TA_, and *C*_TB_ values. Defining these parameters enables the calculation of the surface charge densities of the GMB cell membranes from Equation (3) for comparison to experimental data.

### 4.3. Statistical Analysis

The cell-based experiments were repeated at least three times, and data are presented as the mean ± standard deviation (SD). Statistica Data Miner (Dell, Round Rock, TX, USA) was used to perform statistical analysis. One-way analysis of variance was carried out for comparisons between control and treated groups. The half maximal inhibitory concentration (IC_50_) values were calculated using the GraphPad Prism 5 software (GraphPad Software, Inc., San Diego, CA, USA). A *p* value less than 0.05 was set for statistical significance.

## 5. Conclusions

In this study, both experimental and theoretical approaches have been employed to describe surface charge density changes of GBM cell membranes upon cinnamic and ferulic acids treatment. Determined values, such as electric charge or concentrations of functional acidic and basic groups on treated and untreated cancer cell membranes may be used in the description of the phenomena existing in membranes of living cells and their biophysical studies. Therefore, the significance of such studies can be particularly valuable for modern medical and pharmacological applications and can contribute to the progress in translating chemical theories into clinical practice. Thus, providing information on the mechanisms existing in biological membranes exposed to various pharmacological agents may help to unravel potential physicochemical dependencies determining membrane-permeability and bioavailability of drug candidates.

## Figures and Tables

**Table 1:** Reported anti-cancer activities of cinnamic and ferulic acids.

Phenolic Acid	Type of Cancers	Cell Lines Used	Reported IC_50_^1^ [mmol/dm^3^]	Reference
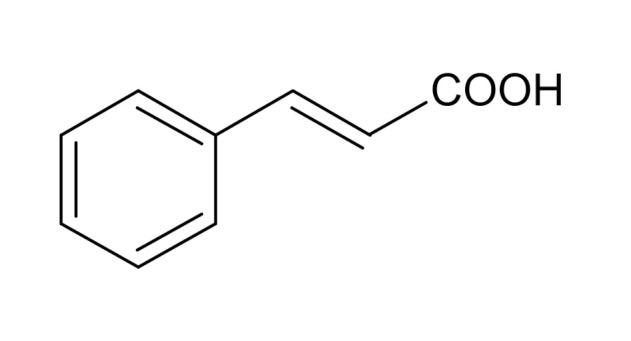 Cinnamic acid (CA)	Glioblastoma	A172	4.50 ± 0.50	[19]
		U251	4.00 ± 1.00	[19]
	Melanoma	HT-144	2.40	[20]
		MEL 1011	2.40 ± 1.00	[19]
		AS75(M)	1.00 ± 0.20	[19]
		SKMEL28	2.50 ± 0.10	[19]
	Prostate	PC3(M)	2.70 ± 0.50	[19]
		Du145	4.00 ± 0.50	[19]
		LNCaP	1.90 ± 1.00	[19]
	Colon	HT-29	1.00	[21]
		Caco-2	4.00–5.00	[22]
		HCT 15	0.80	[23]
	Nasopharyngeal	CNE2	−−− ^2^	[24]
	Lung	A549	1.20 ± 0.10	[19]
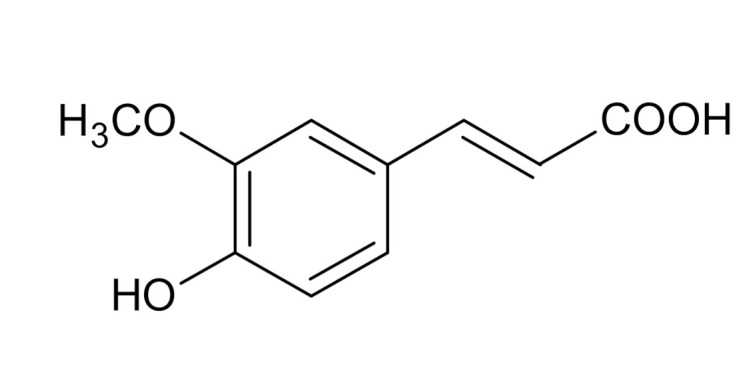 Ferulic acid (FA)	Prostate	PC-3	0.30	[25]
		LNCaP	0.50	[25]
	Colon	HT-29	1.21 ± 0.07	[26]
		LOVO	0.69 ± 0.10	[26]
		HT29-D4	−−− ^2^	[27]
		HCT-8	1.12 ± 0.03	[26]
	Lung	A549	−−− ^2^	[27]
	Breast	EMT-6	0.87 ± 0.09	[26]
	Pancreatic	MIA-Pa-Ca-2	0.50	[28]
	Lymphoma	SW-620	0.98 ± 0.12	[26]
	Osteosarcoma	143B	0.60	[29]

^1^ Data are expressed as IC_50_, the concentration necessary to reduce survival to 50% of the value in untreated cells. ^2^ IC_50_ value is not given.

**Table 2:** Effect of cinnamic and ferulic acids on the acidic and basic functional groups concentrations and associations constants with H^+^ and OH^−^ ions of glioblastoma cell lines.

Compound	System	Parameters			
		*C*_TA_	*C*_TB_	*K*_AH_	*K*_BOH_
		(10^−6^ mol/m^2^)	(10^−6^ mol/m^2^)	(m^3^/mol)	(10^7^ m^3^/mol)
CA	LN-229 (24 h)	4.18 ± 0.07	1.16 ± 0.07	194.30 ± 1.12	7.39 ± 0.09
	+1.0 mmol/dm^3^	3.39 ± 0.04	1.37 ± 0.07	208.10 ± 1.14	5.22 ± 0.08
	+3.5 mmol/dm^3^	2.75 ± 0.10	1.56 ± 0.04	284.00 ± 1.09	3.85 ± 0.09
	LN-229 (48 h)	4.04 ± 0.05	1.11 ± 0.04	175.00 ± 1.15	9.24 ± 0.04
	+1.0 mmol/dm^3^	3.51 ± 0.10	1.24 ± 0.03	308.20 ± 1.13	6.42 ± 0.17
	+3.5 mmol/dm^3^	2.73 ± 0.06	1.45 ± 0.04	343.00 ± 1.22	4.30 ± 0.07
FA	LN-229 (24 h)	4.18 ± 0.07	1.16 ± 0.07	194.30 ± 1.12	7.39 ± 0.09
	+1.0 mmol/dm^3^	3.67 ± 0.12	1.33 ± 0.05	270.40 ± 1.05	5.72 ± 0.10
	+5.0 mmol/dm^3^	2.59 ± 0.08	1.51 ± 0.05	336.10 ± 1.08	4.56 ± 0.14
	LN-229 (48 h)	4.04 ± 0.05	1.11 ± 0.04	175.00 ± 1.15	9.24 ± 0.04
	+1.0 mmol/dm^3^	3.37 ± 0.07	1.35 ± 0.03	208.00 ± 1.07	6.74 ± 0.15
	+5.0 mmol/dm^3^	2.44 ± 0.04	1.44 ± 0.03	311.50 ± 1.18	3.04 ± 0.14

**Table 3:** Drug-like properties of CA and FA.

Compound	*M*_WT_^1^	*c*log*P*^2^	HBA ^3^	HBD ^4^	RB ^5^
CA	148.16	1.98 [44]	2 [45]	1 [45]	2 [45]
FA	194.18	1.42 [44]	4 [45]	2 [45]	3 [2]

^1^ molecular weight, ^2^ calculated n-octanol/water partition coefficient, ^3^ hydrogen bond acceptor groups, ^4^ hydrogen bond donor groups, ^5^ rotable bonds (RBs).

## Supplementary Materials

Supplementary materials can be found at https://www.mdpi.com/1422-0067/21/18/6972/s1. Figure S1 Dose-response curves for (a) CA and (b) FA.

## Abbreviations

CA	Cinnamic acid
FA	Ferulic acid
GBM	Glioblastoma multiforme
BBB	Blood–brain barrier
ELS	Electrophoretic light scattering
σ	Surface charge density
*C*_TA_	Concentration of functional acidic groups
*C*_TB_	Concentration of functional basic groups
*K*_AH_	Association constant with hydrogen ions
*K*_BOH_	Association constant with hydroxyl ions
*M*_WT_	Molecular weight
*c*log*P*	Calculated n-octanol/water partition coefficient
HBA	Hydrogen bond acceptor groups
HBD	Hydrogen bond donor groups
RB	Rotable bonds
