# Supplementary figures and images for: Monitoring of the Surface Charge Density Changes of Human Glioblastoma Cell Membranes upon Cinnamic and Ferulic Acids Treatment
Source: Int J Mol Sci. 2020 Sep 22;21(18):6972. doi: 10.3390/ijms21186972 (PMC7555054; doi:10.3390/ijms21186972)

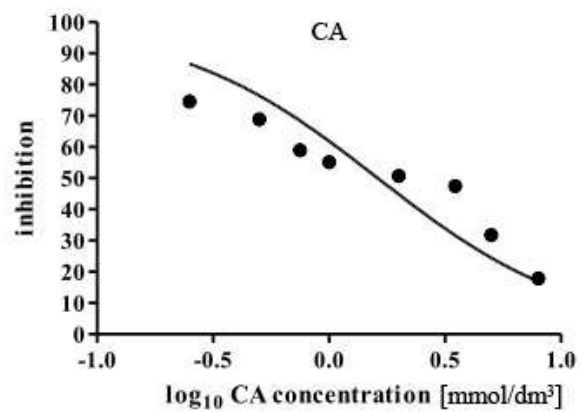

(a)

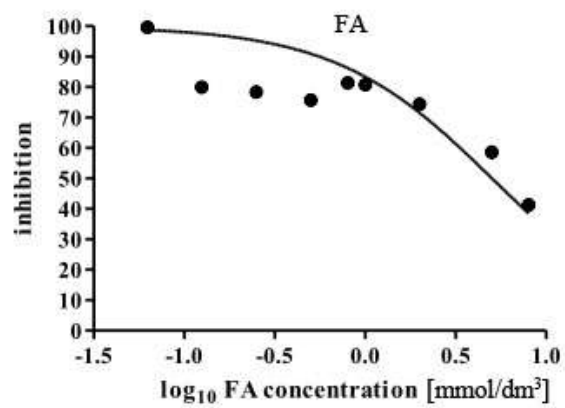

(b)

S1. Dose-response curves for (a) CA and (b) FA.

Supplement: Supplementary file 1 [file ijms-21-06972-s001.pdf]
